# Relationship between prevalence and severity of exercise-induced pulmonary hemorrhage and environmental factors

**DOI:** 10.1093/jvimsj/aalag052

**Published:** 2026-03-29

**Authors:** Erin Pinnell, Sierra Shoemaker, Yuan Wang, Yanan Tang, Debra Sellon, Renaud Leguillette, Jenifer Gold, Macarena Sanz, Warwick M Bayly

**Affiliations:** Veterinary Clinical Sciences, Washington State University, Pullman, WA 99164, United States; Veterinary Clinical Sciences, Washington State University, Pullman, WA 99164, United States; Veterinary Clinical Sciences, Washington State University, Pullman, WA 99164, United States; Department of Mathematics and Statistics, Washington State University, Pullman, WA 99164, United States; Veterinary Clinical Sciences, Washington State University, Pullman, WA 99164, United States; Faculty of Veterinary Medicine, University of Calgary – VCDS, Calgary, AB T2N 4Z6, Canada; Veterinary Clinical Sciences, Washington State University, Pullman, WA 99164, United States; Veterinary Clinical Sciences, Washington State University, Pullman, WA 99164, United States; Veterinary Clinical Sciences, Washington State University, Pullman, WA 99164, United States

**Keywords:** EIPH, risk factors, air quality, ambient temperature

## Abstract

**Background:**

Environmental risk factors could contribute to the development of exercise-induced pulmonary hemorrhage (EIPH) in racing Thoroughbreds.

**Hypothesis/Objectives:**

To identify environmental risk factors that might contribute to differences in EIPH prevalence and severity across 12 Thoroughbred racetracks in the United States.

**Animals:**

Eight hundred fifteen 2-year-old and 122 >2-year-old Thoroughbred racehorses.

**Methods:**

Prospective blinded observational study. Videoendoscopy was performed 30-60 min post-race. Three observers independently assigned an EIPH grade to each videorecording, and prevalence and severity of EIPH were determined. Multivariable logistic regression assessed relationships between EIPH prevalence and severity, respectively, and independent variables including furosemide administration, race distance and surface, and lifetime race number (LRN). *P ≤* .05 was considered significant.

**Results:**

One thousand one hundred ninety-two videorecordings received EIPH severity grades from 12 racetracks in 10 different states and 3 time zones. Ambient temperature (AT) was negatively associated with EIPH prevalence (OR = 0.82, 95% CI, 0.69-0.98; *P* = .03) and severity (OR = 0.79, 95% CI, 0.63-0.99; *P* = .04). Furosemide administration (OR = 0.04, 95% CI, 0.006-0.33; *P* = .002), turf (OR = 0.64, 95% CI, 0.43-0.95; *P* = .027) and all weather (OR = 0.03, 95% CI, 0.004-0.26; *P* = .001) track surfaces, and LRN (OR = 1.07, 95% CI, 1.01-1.12; *P* = .012) were associated with occurrence of EIPH. Two-year-olds racing in bad air (air quality index [AQI] > 100) had more severe EIPH than those racing in AQI < 50 (OR = 2.78, 95% CI, 1.06-7.29; *P* = .04).

**Conclusions and clinical importance:**

AT, AQI, LRN, and surface type might be associated with EIPH prevalence and severity between American racetracks.

## Introduction

Exercise-induced pulmonary hemorrhage (EIPH) is defined as bleeding from the lungs in association with equine exercise and occurs commonly in Standardbred and Thoroughbred racehorses.^1^ Diagnosis is based on post-exercise bronchoalveolar lavage (BAL), tracheoendoscopic examination, or both.^1^^,^^2^ This condition occurs in most horses undergoing high intensity exercise including 2-year-old Thoroughbred racehorses, which have a prevalence of 74% based on post-race endoscopic examination.^3^ The fundamental cause of EIPH is related to the development of high transmural pressures across the pulmonary capillary-alveoli interface which result in disruptions in the structural integrity of this interface.^4^ Peak inspiratory intrapleural pressure is a key determinant of transmural pressure and the presence of any environmental factor that could induce an increase in this peak pressure by narrowing airways might contribute to EIPH’s occurrence, increase the severity of EIPH, or contribute to both EIPH occurrence and severity.

Several horse-, track-, and race-related risk factors have been associated with higher prevalence and severity of EIPH.^5–7^ Some of these factors include race type (steeplechase versus flat races), race distance, topographical altitude, sex, age, and the cumulative number of lifetime races (LRN) in a horse’s career.^8^ The effects of environmental conditions on the progression of EIPH have not been extensively studied. Previous studies in Australia and Japan found that ambient temperature (AT) was negatively correlated with EIPH prevalence.^5^^,^^7^ Horses racing at low AT (<20 °C) are nearly 2 times greater risk of experiencing EIPH, and lower AT is associated with EIPH grades ≥1.^5^ Other studies report that lower AT was associated with EIPH grades ≥2.^5^^,^^7^ However, there were no other environmental factors that appeared to be associated with EIPH. Another recent report noted differences in prevalence and severity of EIPH among 12 different Thoroughbred racetracks throughout the United States,^3^ but the reasons for these geographical differences were not identified.

The purpose of this study was to evaluate environmental risk factors that could contribute to an association between racetrack location and the prevalence and severity of EIPH in the United States. We hypothesized that a poor air quality index (AQI) and low ATs would both be associated with increased prevalence and severity of EIPH in Thoroughbred racehorses and that they would be more common at some tracks than others.

## Materials and methods

The study was approved by the Washington State University Institutional Animal Care and Use Committee protocol (ASAF#6843).

### Study design

This was a blinded observational study involving 2-year-old Thoroughbreds, including some stakes horses, and older stakes horses that raced at 12 American racetracks identified as A-L (Table 1), between August 2020 and August 2021. These tracks were in 10 different states, extending from the east coast to the west coast, and from northern United States near the Canadian border to southern California and Florida. All were located at altitudes <300 m. The evaluated cohort consisted of 2-year-olds racing in Maryland in 2020 where post-race tracheoendoscopy of 2-year-olds was legislatively mandated (“mandated”), horses that were routinely examined tracheoendoscopically by their veterinarians after racing (“routine”), and others that were voluntarily enrolled in the study by their trainers (“requested”). Tracheoendoscopy was performed by the horses’ veterinarians 30-60mins post-race and a videorecording was made of the examination. Some horses at several of the tracks received 250 mg furosemide IV 4 h before racing.

**Table 1:** The distribution of EIPH grades (grade 0-4) across 1192 videoendoscopy recordings of Thoroughbred 2-year-olds and stakes quality racehorses >2-years-old at 12 racetracks (A-L) located across the United States.

**Track/EIPH grade**	**0**	**1**	**2**	**3**	**4**	***n***
**A**	26	20	28	8	2	84
**B**	8	34	23	10	1	76
**C**	13	10	10	2	0	35
**D**	6	13	9	3	1	32
**E**	3	14	10	1	2	30
**F**	60	52	80	32	7	231
**G**	34	21	8	3	0	66
**H**	23	15	6	6	0	50
**I**	177	194	75	18	3	467
**J**	18	23	9	2	0	52
**K**	17	4	2	1	0	24
**L**	8	10	17	8	2	45

### Detection and grading the severity of EIPH

Videorecordings of the tracheoendoscopic examinations were uploaded to a secure website by the horses’ veterinarians. All information identifying each horse, its trainer, and the track on which it raced was removed and replaced by a randomly generated number. Each videorecording was then sent to 3 veterinarians with experience evaluating EIPH severity for assignment of a grade on a scale of 0-4.^9^ If there was no unanimity regarding the EIPH grade for a particular videorecording, that assigned by 2 of the veterinarians was accepted. If each veterinarian assigned a different grade to a video or it was deemed to be of insufficient quality to allow a grade to be assigned, the video was deleted.

### Recorded variables

Environmental variables that were considered included AQI, AT, housing type (brick, cement, tent, or wood), interior- or exterior-facing horse stall location in a barn, type of bedding (straw or wood shavings), and residency of horses in a racetrack barn (resident) compared to non-racetrack locations (non-resident). Additionally, LRN, the use of furosemide before racing, race distance categorized as <1300 m (short), 1300-1500 m (middle), and >1500 m (long), type of endoscopy service (mandated, routine, requested) and track surface (dirt, turf, all-weather) were included as variables in the multivariable regression analysis. The date and time of day of each race for each horse from which a videorecording was received and whether it received furosemide 4 h before the race were obtained from the online racing database, Equibase®. AQI for the county in which each track was located was gathered from the United States Environmental Protection Agency’s website (www.epa.gov) for the time of day of each race. The AT for the time of day of each race was recorded using Weather Underground’s website (www.wunderground.com). Individual trainers of a subgroup of horses provided information regarding the type of stall in which the horse was housed (building materials and exterior or interior facing stall), and if the horse was a resident or non-resident. Trainers’ input was also sought regarding the period in which a horse resided, trained, and raced at a specific racetrack, and 14-day AQI means were calculated for horses that had been residents at that track for ≥14 days. Information regarding the horses’ bedding was obtained for the 2-year-olds that raced in Maryland.

### Statistical analyses

The overall prevalence of EIPH and distribution of EIPH grades of severity was tabulated for each racetrack and for the entire study sample. The distribution of EIPH was also tabulated for each living condition variable. Simple descriptive results were determined for AQI and AT for each EIPH grade. Univariate analysis evaluated the association between ordinal EIPH grade and each of the above listed independent variables. Pearson’s χ-square test of independence was applied to the living condition factors which were categorical variables. For numerical variables, the ANOVA F test was used, and Tukey’s simultaneous confidence interval post-hoc test was applied for pairwise comparisons when the F statistic was significant. Residual plots and QQ plot were used to check for variance of equality. The Shapiro–Wilk test of normality was applied to check for possible departures from the normality of statistical assumptions.

Mixed-effects multivariable regression analysis was used to determine significant associations between environmental variables (AT and AQI) and track location and to control for confounding between the sets of independent variables. Two outcomes of EIPH prevalence (grade = 0 or grade ≥ 1) and EIPH severity (grade < 3 or grade ≥ 3) were considered in this analysis. Separate logistic regression models were implemented for each outcome. Random effects for tracks and horses were considered to account for repeated starts within tracks, variations across tracks, and repeated races within horses. Random effects for AQI and AT were also investigated. The living condition variables, race location, endoscopy service type, LRN, age, pre-race furosemide administration status, and race surface and distance categories were included in the mixed-effects model. The AQI was reported as a categorical variable: good (<50), moderate (50-100), and bad (>100) air quality. Ambient temperatures were standardized with mean = 0 and SD = 1 to improve model estimation convergence. Living condition variables were evaluated based on the *P*-value because they were only available for a subset of racetracks with various sample sizes, thus making the Akaike information criterion (AIC) type of model selection inappropriate. Moreover, because LRN and age were highly correlated, we considered models with age, LRN, and both. The final model was selected based on AIC. Model fitness was assessed by checking the deviance residuals and the area under the receiver operating curve. The multivariable regression analysis was first performed on all horses, and then only the 2-year-olds and the results compared. All analyses were performed using R (Studio Version 2025.05.1). Significance was deemed to exist when *P* ≤ .05.

## Results

Results are reported as the mean ± SD, unless otherwise noted. The EIPH grade was determined for 1192 videorecordings from 815 two-year-olds (including 97 stakes horses), and 122 >2-year-old stakes horses. There were 1055 videos from 2-year-olds. The mean age of horses >2-years-old was 3.9 ± 1.2 years. Videos were obtained after 275 races on turf, 893 races on dirt, and 24 races on an all-weather surface. Of the 1192 videos, 559 were recorded after races of <1300 m, 185 were from races of 1300-1500 m, and 448 videos were associated with races >1500 m. The mean LRN for all horses was 3.3 ± 4.0 races. For 2-year-olds, it was 2.4 ± 1.4 with a maximum of 9, and for horses >2-years-old was 10.2 ± 8.3 races (maximum = 49). Furosemide was approved for use at 5 tracks and was administered before 145 race starts (2-year-olds: 117; >2-years-old: 28). There were 520 mandated videorecordings from Maryland, 520 routine ones, and 152 obtained on-request. EIPH (grade ≥ 1) was observed in 799 (67.1%) of the videorecordings, with 410 videos (34.3%) reported as grade 1 EIPH, 277 videos (23.5%) reported as grade 2 EIPH, 94 videos (7.7%) reported as grade 3 EIPH, and 18 videos (1.6%) reported as grade 4 EIPH. Median EIPH grades for all horses and for 2-year-olds were the same: 1, interquartile range (IQR) 0-2.

Ambient temperature ranged from −0.6 to 34 °C (19.4 ± 7.2; median 19.4, IQR 13.3-25.6). In one location, the race time AT ranged from −0.6 to 7 °C over a 10-day period. The distance of the AQI monitoring station from a racetrack was 16 ± 10 km with the longest distance being 34 km. The AQI ranged from 12 to 120 (40 ± 18; median 35, IQR 28-46). At one racetrack AQI was >100 for a week of racing. Fourteen-day AQI means were obtained for 39 horses that had been residents at one of 3 racetracks for ≥14 days and ranged from 30 to 56. These means were not different to the race day AQI (20-62) for the same horses.

### Univariate analysis

Univariate analysis revealed a significant difference in EIPH grade between the 12 track locations (*P* < .001). The type of material from which 571 of the horses’ barn stalls were constructed was obtained from 7 tracks and found to have no association with EIPH grade (*P* = .59). Information regarding the placement of a horse’s stall within the barn (interior or exterior door opening) was gathered for 149 horses from 4 track locations. Because the median and IQR of EIPH scores were identical between groups (1; IQR 0-2), we further evaluated EIPH prevalence (grade > 0 versus grade = 0). There was a significant association between EIPH prevalence and stall placement (*P* < .001). Horses with an interior placement had markedly lower odds of EIPH occurring compared with those with an exterior placement (OR = 0.18, 95%CI, 0.08-0.40). However, the majority of horses housed in exterior facing stalls were housed at tracks with a lower AT (58.0 ± 9.5°C) whereas those at tracks with a higher AT had most horses living in interior-facing stalls (AT 22.1 ± 6.1°C).

Data regarding whether a horse was a resident or non-resident at the track were obtained in conjunction with 673 post-race tracheoendoscopic examinations. The distribution of EIPH grades differed by residency status (*P* = .03). Shipped-in horses had lower odds of exhibiting any endoscopic evidence of EIPH when compared with resident horses (OR = 0.61, 95%CI, 0.44-0.85). Information regarding the horses’ bedding type was obtained in conjunction with 565 videoendoscopic recordings and no significant association found between the use of straw or shavings and EIPH prevalence (*P* = .62).

### Multivariable logistic regression analyses

All 1192 assigned EIPH grades from the 12 tracks were included in the multivariable analyses for all horses. An additional analysis was independently performed on the 1055 EIPH grades for 2-year-olds to reduce potential age-related bias. A random effect for tracks was included to account for variations across tracks in both analyses. For analyses related to EIPH prevalence, a random horse effect was also included for possible within-horse correlation. For EIPH severity analysis, only 15 out of >900 horses showed any within-horse change across observations. The random horse effect was weakly identified and did not add significantly to the model. Consequently, each observation was treated as an independent sampling unit. The initial results for all these analyses revealed that there were no significant interactive effects between AQI, housing, bedding, stall placement, and track resident vs non-resident status. However, there were significant random track effects, indicating that EIPH prevalence and severity differed between tracks. To further investigate these differences among track locations, LRN was included in the mixed effects multiple regression model based on the AIC calculations.

When LRN was included in the model, the variance of the random track effects dropped from 0.31 to 0.21, thus explaining some of the location-related differences. The LRN a horse had competed in was positively related to both EIPH prevalence (*P* = .01) and severity (*P* < .001). Because there was a significant association between LRN and EIPH prevalence and severity, the model including LRN was utilized to determine any associations between environmental variables and EIPH prevalence or severity.

### Multivariable regression analysis results for all horses

In the mixed-effects model for EIPH prevalence, AT, furosemide use, LRN, racetrack surface, and endoscopy service type were significant predictors of EIPH (Table 2). There was a significant difference in AT across all 12 racetrack locations (Figure 2). Higher AT was associated with reduced odds of EIPH (*P* = .03). Horses administered furosemide had lower odds of EIPH compared with untreated horses (*P* = .002). LRN was positively associated with prevalence (*P* = .001). Compared with dirt tracks, races on turf were associated with lower odds of EIPH (*P* = .027), while all-weather surfaces showed a much stronger protective association, with lower EIPH prevalence (*P* = .001). Horses examined during mandated and routine endoscopy services had lower odds of EIPH compared with requested endoscopies (*P* = .002 and *P* = .005, respectively). Air quality and race distance were not significantly associated with prevalence.

**Table 2:** Mixed-effects model results (odds ratios, 95% CI, and *P-*values) from the multivariable regression analysis for EIPH prevalence from 1192 videorecordings of post-race tracheoendoscopic examinations of 937 Thoroughbred racehorses.

**Variable**	**Odds ratio**	**95% CI (lower)**	**95% CI (upper)**	***P*-value**
**AQI <50**	1	NA	NA	NA
**AQI 50-100**	1.22	0.84	1.79	.300
**AQI >100**	1.99	0.49	8.15	.337
**AT**	0.82	0.69	0.98	.030
**Furosemide (N)**	1	NA	NA	NA
**Furosemide (Y)**	0.04	0.006	0.33	.002
**Lifetime starts**	1.07	1.01	1.12	.012
**Short distance**	1	NA	NA	NA
**Middle distance**	1.05	0.68	1.63	.820
**Long distance**	1.05	0.75	1.46	.777
**Dirt surface**	1	NA	NA	NA
**Turf surface**	0.64	0.43	0.95	.027
**All weather surface**	0.03	0.004	0.26	.001
**Requested endoscopy**	1	NA	NA	NA
**Routine endoscopy**	0.04	0.004	0.37	.005
**Mandated endoscopy**	0.02	0.002	0.25	.002

Furosemide (Y) indicates the use of furosemide before racing.

**Figure 1:**
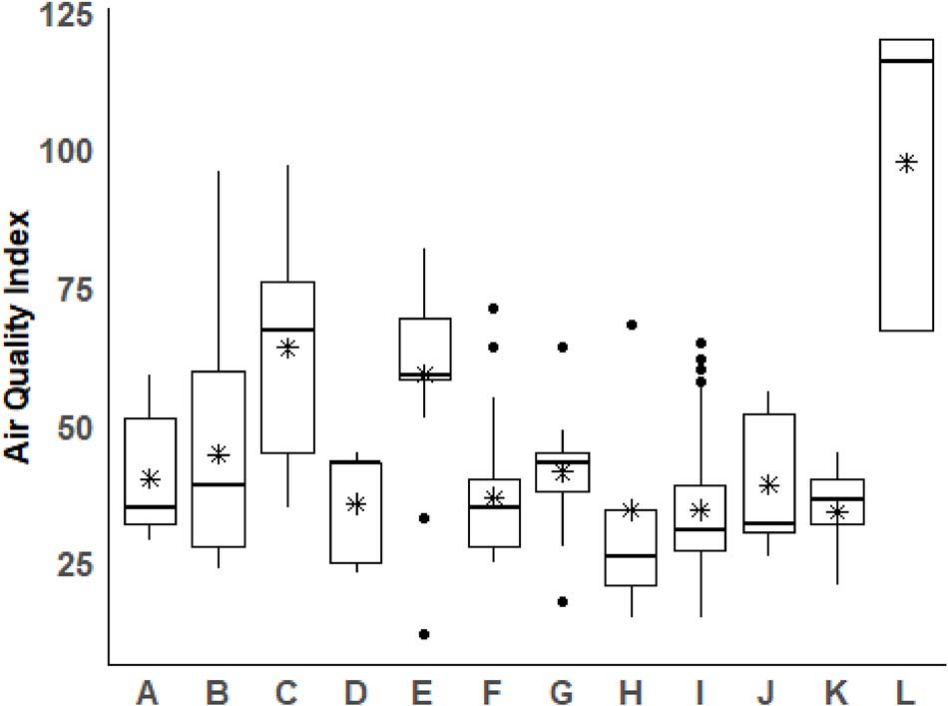
The relationship between air quality index (AQI) and racetrack location. There was a significant difference in AQI among the 12 tracks (*P* < .001). Within each box, the horizontal black lines denote median values; boxes extend from the 25th to the 75th percentile of each track’s distribution of values. Vertical extending lines denote adjacent values. The stars in each boxplot represent the mean AQI.

The model for EIPH severity indicated associations with AT, LRN, and track location (Maryland vs other states; Table 3). Despite AQI being significantly different among the 12 racetracks (*P* < .001, Figure 1), the odds that horses racing in bad quality air (AQI > 100) would have more severe EIPH compared with those racing in good air quality (AQI < 50) were not different (*P* = .09) and moderate air quality was not a significant determinant of EIPH severity. Higher AT was negatively associated with EIPH severity (*P* = .04), and LRN remained strongly associated with greater severity (*P* < .001). Horses at Maryland tracks had significantly lower odds of severe EIPH compared with other tracks (*P* < .001). Furosemide use, endoscopy service category, and race distance were not significantly associated with EIPH severity.

**Table 3:** Mixed-effects model results (odds ratios, 95% CI, and *P-*values) from the multivariable regression analysis for EIPH severity from 1192 videorecordings of post-race tracheoendoscopic examinations of 937 Thoroughbred racehorses.

**Variable**	**Odds ratio**	**95% CI (lower)**	**95% CI (upper)**	***P*-value**
**AQI <50**	1	NA	NA	NA
**AQI 50-100**	1.27	0.78	2.07	.343
**AQI > 100**	2.25	0.89	5.73	.088
**AT**	0.79	0.63	0.99	.044
**Furosemide (N)**	1	NA	NA	NA
**Furosemide (Y)**	0.27	0.02	3.28	.303
**Lifetime starts**	1.09	1.05	1.13	.000
**Short distance**	1	NA	NA	NA
**Middle distance**	1.32	0.71	2.44	.380
**Long distance**	1.12	0.68	1.85	.656
**Dirt surface**	1	NA	NA	NA
**Turf surface**	1.13	0.66	1.92	.651
**All weather surface**	0.41	0.03	5.47	.499
**Requested endoscopy**	1	NA	NA	NA
**Routine endoscopy**	0.49	0.04	6.04	.578
**Mandated endoscopy**	0.16	0.01	2.00	.154

Furosemide (Y) indicates the use of furosemide before racing.

### Multivariable regression analysis results for 2-year olds

Among 2-year-olds, AT, furosemide use, and racetrack surface type were significant predictors of EIPH prevalence (Table 4). Higher AT was associated with reduced odds of EIPH (*P* = .02). Horses administered furosemide had lower odds of exhibiting EIPH than untreated horses (*P* = .01). Races conducted on all-weather surfaces showed a strong protective effect relative to dirt (*P* = .002). There was no difference in the odds ratio of EIPH occurring between dirt and turf tracks (*P* = .13). Lifetime race number was not a predictor of EIPH prevalence in 2-year-olds (*P* = .74). Air quality, race distance, and track location were not significantly associated with EIPH prevalence. The provision of mandated (*P* = .009; Table 4) and routine (*P* = .02) post-race endoscopy examinations was associated with a lower EIPH prevalence than endoscopy provided on-request (Table 4).

**Table 4:** Mixed-effects model results (odds ratios, 95% CI, and *P-*values) from the multivariable regression analysis for EIPH prevalence from 1055 videorecordings of post-race tracheoendoscopic examinations of 815 2-year-old Thoroughbred racehorses.

**Variable**	**Odds ratio**	**95% CI (lower)**	**95% CI (upper)**	***P*-value**
**AQI <50**	1	NA	NA	NA
**AQI 50-100**	1.25	0.85	1.84	.258
**AQI >100**	2.15	0.54	8.57	.280
**AT**	0.81	0.68	0.97	.023
**Furosemide (N)**	1	NA	NA	NA
**Furosemide (Y)**	0.06	0.007	0.57	.014
**Lifetime starts**	1.02	0.91	1.14	.736
**Short distance**	1	NA	NA	NA
**Middle distance**	1.14	0.73	1.79	.567
**Long distance**	1.07	0.74	1.53	.720
**Dirt surface**	1	NA	NA	NA
**Turf surface**	0.72	0.47	1.11	.137
**All weather surface**	0.04	0.006	0.32	.002
**Requested endoscopy**	1	NA	NA	NA
**Routine endoscopy**	0.059	0.005	0.63	.019
**Mandated endoscopy**	0.038	0.003	0.44	.009

Furosemide (Y) indicates the use of furosemide before racing.

EIPH severity in 2-year-olds was associated with AQI, AT, and track location (Table 5). Horses racing when AQI >100 had higher odds of severe EIPH compared to those racing in good quality air (*P* = .04), whereas moderate air quality was not a significant factor. Higher AT was associated with less severe EIPH. (*P* = .02). Two-year-olds racing in Maryland had reduced odds of severe EIPH relative to other tracks (*P* < .001). In contrast to the all-horse model, LRN was not associated with EIPH severity in 2-year-olds. Furosemide use, race distance, and endoscopy service type were also not significant contributing determinants of EIPH severity.

**Table 5:** Mixed-effects model results (odds ratios, 95% CI, and *P-*values) from the multivariable regression analysis for EIPH severity from 1,055 videorecordings of post-race tracheoendoscopic examinations of 815 2-year-old Thoroughbred racehorses.

**Variable**	**Odds ratio**	**95% CI (lower)**	**95% CI (upper)**	***P*-value**
**AQI <50**	1	NA	NA	NA
**AQI 50-100**	1.40	0.83	2.36	.202
**AQI > 100**	2.78	1.06	7.29	.038
**AT**	0.74	0.57	0.95	.021
**Furosemide (N)**	1	NA	NA	NA
**Furosemide (Y)**	0.34	0.02	5.00	.428
**Lifetime starts**	0.95	0.80	1.12	.520
**Short distance**	1	NA	NA	NA
**Middle distance**	1.48	0.78	2.81	.235
**Long distance**	1.21	0.67	2.17	.527
**Dirt surface**	1	NA	NA	NA
**Turf surface**	0.98	0.51	1.86	.941
**All weather surface**	0.54	0.04	7.56	.650
**Requested endoscopy**	1	NA	NA	NA
**Routine endoscopy**	0.61	0.04	9.18	.722
**Mandated endoscopy**	0.22	0.01	3.31	.272

Furosemide (Y) indicates the use of furosemide before racing.

## Discussion

This study examined contributing factors to the prevalence and severity of EIPH across various geographic regions in the United States. Although previous research had documented significant differences in EIPH prevalence among Thoroughbreds racing at 12 racetracks nationwide, the underlying causes of these variations had not been explored.^3^ The present study affirmed that finding and investigated specific environmental factors that might explain those differences. Several significant associations were identified.

Four multivariable analyses were conducted to ensure that any association of environmental factors, the horses’ ages, or a combination of both, on EIPH prevalence or severity were accounted for. AT was the only factor associated with both EIPH prevalence and severity in both cohorts of horses. Lower ATs increased the odds of EIPH occurring and were negatively related to EIPH severity. The result regarding EIPH prevalence was comparable with a study of 744 Thoroughbreds competing at several racetracks in a large metropolitan city which found that EIPH was about 1.9 times more likely to occur with AT <20 °C.^6^ A study of Standardbred horses racing in Quebec when the ambient temperature was as low as −15 °C identified a significant correlation between EIPH occurrence and lower AT.^10^ However, the findings in that study were confounded by the fact that there was also a positive association between air pollution and EIPH (AQI was not measured) as the high density of the freezing air concentrated the pollutants.

Although a causative link between low AT and EIPH has yet to be identified it is most likely due to lower airway inflammation induced by the inhalation of cool/cold air, similarly induced exercise-induced bronchoconstriction (EIB), or both. The direct effects of inhaling cool/cold air on equine airway mechanics during strenuous exercise have not been thoroughly evaluated and the possibility that this activity causes exercise-induced bronchoconstriction of airway smooth muscle and increased airway resistance cannot be ruled out. In people, EIB usually occurs shortly after the cessation of exercise rather than during it.^11^ In horses, inhaling subfreezing air (−5 °C) during submaximal exercise had no acute negative effects on airway resistance, reactance, or impedance.^12^ Conversely, there have been multiple studies linking inhalation of cold air with extensive inflammation of the lower airways and these have been extensively reviewed,^13^ and this appears to be the most likely basis for the significant association between AT and EIPH found in this study.

This contention is supported by the finding that breathing of cool (4 °C) unconditioned air by strenuously exercising horses resulted in extensive mucosal damage and inflammation of their peripheral lower airways.^14^ This was presumably because, as a consequence of the high respiratory frequency, minute ventilation, and peak inspiratory airflow rates that characterize such exercise, the large volumes of inspired cold air exceeded the capacity of the normal warming and humidification mechanisms of the upper airways, and penetrated to the furthest reaches of the lower airways, resulting in airway desiccation and inflammation.^14^ Repeated hyperventilation of cold air by dogs is associated with extensive mucosal damage, including intraluminal exudate and leakage of blood from the bronchial vasculature into the airways.^15^ These findings raise the possibility that part of the reason for the detected increase in severity of EIPH associated with lower AT could be hemorrhage from the bronchial circulation. While EIPH typically originates from the pulmonary capillaries due to changes in their permeability secondary to large increases in the capillary-alveolar transmural pressure,^16^ this does not rule out the possibility that blood of bronchial vascular origin could be contributing to the increased volume of blood visualized in post-race videoendoscopic recordings from horses that have just competed in low AT conditions.

**Figure 2:**
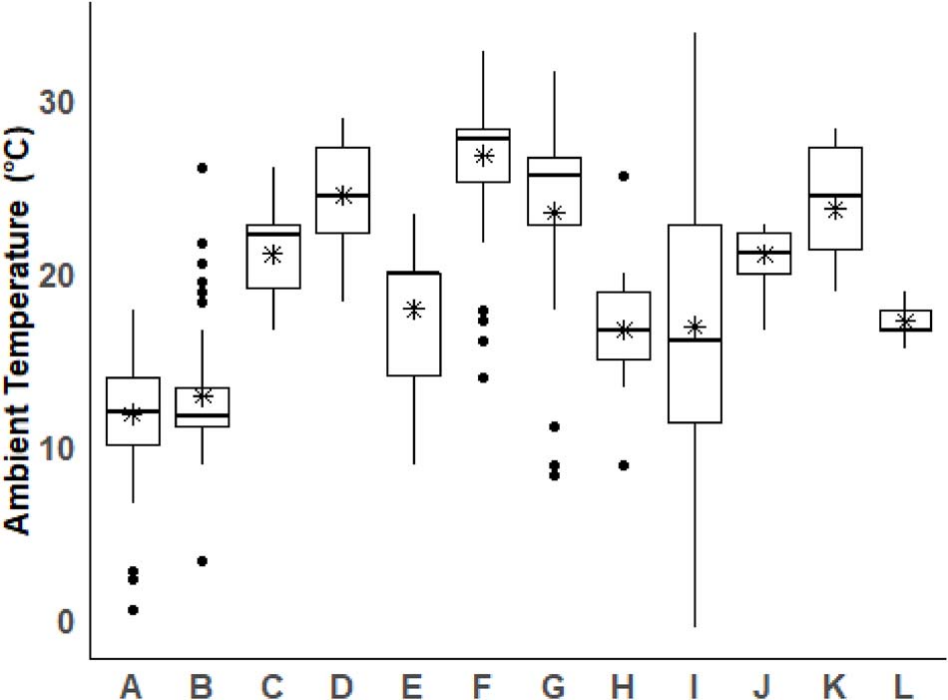
The relationship between ambient temperature (AT) and track location. There is a significant difference in AT among the 12 racetracks (*P* < .001). Within each box, horizontal black lines denote median values; boxes extend from the 25th to the 75th percentile of each track’s distribution of values. Vertical extending lines denote adjacent values. The stars in each boxplot represent the mean AT.

Although the period in which horses were exposed to low AT could not be determined and probably varied, it is also likely that some horses were exposed to low temperatures over lengthy periods as reflected by the observation that the highest maximum temperature over a 10-day period at 1 track was 7 °C (track L, Figure 2). When it is considered that many horses were also exercising and breezing under these conditions, it appears likely that many of these horses developed some degree of lower airway inflammation because of these activities.

Horses with exterior facing stalls had more severe EIPH than those with interior facing stalls. While this was counterintuitive from an AQI perspective, the result could be related to the fact that the majority of exterior facing stalls housing horses in this study were at racetracks where the AT was lowest. Although the AT of exterior and interior facing stalls at the same track were not recorded, it could be hypothesized that in situations in which the AT was <10 °C, horses living in exterior facing stalls might have been more exposed to the wind and colder ambient temperatures than those living in the interior of the barn. Such horses might be more prone to the negative effects of persistently inhaling cool/cold air on airways and more susceptible to EIPH as a result.

Although the AQI was different across the 12 racetracks it did not have a consistent effect on EIPH prevalence in either of the tested cohorts of horses. Its effect was most marked in 2-year-olds for which racing in bad air quality conditions was associated with greater EIPH. Locations with the highest AQI (eg, track L, Figure 1) demonstrated greater severity of EIPH and horses racing in air with AQI >100 had a higher chance of having severe EIPH (grade 3 or 4) than horses racing in good quality air. A previous study based in one city was unable to find an association of EIPH with air quality,^5^ in contrast to the current study in which there was a wider range of AQI recorded due to the involvement of 12 racetracks from widely differing locations within the United States. Two other studies identified a lower level of performance, including decreased speed, at high concentrations of air pollutants, particularly with higher concentration of fine particulate matter, but did not evaluate their effect on EIPH.^17^^,^^18^

The possibility that bad AQI induced lower airway inflammation which in turn resulted in more severe EIPH cannot be discounted although we could find no reports that conclusively link equine inflammatory airway disease like mild-to-moderate asthma (MEA) to the prevalence or severity of EIPH. Higher particulate matter and bad AQI are linked to MEA and reduced performance.^18–20^ Another hallmark of MEA is increased mucus production^21^ and a reduction in racing performance.^18^^,^^19^^,^^22^ However, none of these studies investigated the possible association between increased mucus, decreased performance and EIPH, and 2 other studies found no correlation between post-racing tracheal mucus score and EIPH severity^23^ despite the fact that one of them found that mucus score was positively associated with race day AQI, but not the 14-day AQI for the period leading up to race day.^24^ Because MEA tends to be a chronic/cumulative environmentally-linked inflammatory condition (rather than acute) efforts were made in this study to record the mean 14-day AQI before a race day. The results were disappointing because of the limited number of horses for which this information could be collected due to unexpectedly high rates of horse movement. All the 14-day AQI means that were collected fell into the category of good air quality (AQI < 50) and did not differ from the AQI recorded on race day.

Possibly the best indication of a possible association between MEA and EIPH was the finding in this study that grades of EIPH severity in resident horses were higher than the EIPH grades in non-resident horses. While the AQI at the locations where the non-resident horses were housed and trained was not determined, it is possible that resident horses had more severe EIPH because the AQI at the various track locations was worse than that at the centers where the non-residents trained. Racetracks are typically located in areas of greater populations and road traffic with worse AQI than at training centers as the latter are usually more rurally located with lower density populations and fewer vehicles. A similar difference between residents and shipped-in racehorses in severity of another chronic respiratory tract condition, pharyngeal lymphoid hyperplasia (PLH), was identified in a previous study, with horses that were transported to the racetrack for a race having lower PLH scores than those horses that lived and trained on the racetrack.^25^ While these findings support a possible link between MEA and EIPH, they also suggest that such an association is not clear-cut. If it does exist, it might involve other environmental factors as well as AQI and a large-scale multifactorial study is indicated.

The racetrack surface type and the pre-race administration of furosemide were also associated with EIPH prevalence but not severity, in both cohorts of horses. The reason why a difference in prevalence but not EIPH severity was associated with the different racetrack surfaces was not apparent and warrants further investigation. The finding that horses that were treated with furosemide before racing had lower odds of having EIPH than horses that did not receive furosemide was compatible with previously reports.^26^ However, the result that furosemide did not reduce the severity of EIPH was unexpected given previous reports to the contrary. The most likely explanation is that this was not a crossover study, there were few starts associated with furosemide administration (145, 12% of the total) and only a small percentage of starts associated with severe EIPH (<10%).

Lifetime run number was positively associated with EIPH prevalence and severity in all horses but not the 2-year-olds alone. This was not surprising in view of the results of previous studies which identified that a horse with a higher LRN was more likely to have EIPH and that it was more severe when compared to horses with fewer LRN.^4^ In this study the mean number of lifetime starts by horses >2-years was 5 times greater than that for the 2-year-olds and most starts by an older horse was 40 more than the most by a 2-years-old horse.

Lastly, horses racing at Maryland racetracks where post-race endoscopy was legislatively mandated had lower odds of severe EIPH being detected when compared to the results from other racetracks including those where horses were routinely examined after racing. Additionally, 2-year-olds racing at Maryland racetracks also had reduced odds of severe EIPH being detected when compared to 2-year-olds racing at the racetracks outside that state where some, but not all horses were routinely endoscoped after racing. Of those horses that were not routinely examined, it is likely that only those suspected of having experienced EIPH were examined upon the request of their trainer as reflected by higher prevalence and severity of EIPH in this category of horses.

Topographical altitude was a variable that could have been considered as a factor affecting EIPH in this study as there are reports that racing at altitudes >1400 m might be associated with reductions in the prevalence and severity of EIPH when compared to findings at sea-level.^8^ However, all of the races across the 12 racetracks evaluated were at <300 m altitude, so this variable likely did not play a role in EIPH prevalence and severity and therefore was not considered in the univariate or multivariable regression analyses.

There were several limitations associated with this study. First, the assessment of the influence of the environmental variables was based on post-race tracheoendoscopy results. While this is the preferred method for diagnosing EIPH, its sensitivity is relatively low when compared to that of BAL.^27^ Therefore, the possibility that the results would have been different had BAL been involved in the determination of prevalence cannot be ruled out.

With respect to the role that barn-related environmental factors might have had on the prevalence and severity of EIPH, the lack of information for every horse for which an EIPH grade was assigned was another limitation. More successful collection of this data might have helped determined whether there was a significant association between type of bedding and EIPH prevalence and severity. Being largely limited to the use of race day AQI as well as AT rather than having data that spanned a number of days leading up to a race could have also enabled a more in-depth assessment of their role as risk factors for EIPH.

### Conclusion

Racing in cool to cold AT conditions significantly increased the chances of developing EIPH and it was more severe under these conditions. Other factors that played a role to varying extents in EIPH included AQI, furosemide use, LRN, racetrack surface, and the track’s geographic location. Finally, stall locations in barns appeared to be an additional factor that could be associated with the prevalence and severity of EIPH. Overall, we concluded that there are multiple factors capable of influencing the occurrence and severity of EIPH. Although AT appears to be the major one, how all the factors do or can interact is complex and likely highly variable among American racetracks.

## Off-label antimicrobial declaration

Authors declare no off-label use of antimicrobials.

## Institutional animal care and use committee or other approval declaration

Approved by the Washington State University Institutional Animal care and Use committee, ASAF#6843.

## Abbreviations

AQI: air quality index
AT: ambient temperature
EIB: exercise-induced bronchoconstriction
EIPH: exercise-induced pulmonary hemorrhage
LRN: number of lifetime races
PLH: pharyngeal lymphoid hyperplasia
